# Topographical Patterning of Cell‐Repellent Interfaces for Immune‐Stealth Implantable Electronics via Multiphoton Ablation Lithography

**DOI:** 10.1002/advs.202506482

**Published:** 2025-06-19

**Authors:** Hyunseon Seo, Gwan‐Jin Ko, Sangmin Song, Joong Hoon Lee, Youngmin Seo, Sungkeun Han, Chan‐Hwi Eom, Hyewon Kim, Seongsoo Kim, Kang‐Sik Lee, Yu‐Chan Kim, Hojun Kim, Si‐Eun Moon, Kyungwoo Lee, Seung Hwan Ko, Suk‐Won Hwang, Hojeong Jeon

**Affiliations:** ^1^ Biomaterials Research Center Biomedical Research Division Korea Institute of Science and Technology Seoul 02792 Republic of Korea; ^2^ School of Medicine Sungkyunkwan University Suwon 16419 Republic of Korea; ^3^ KU‐KIST Graduate School of Converging Science and Technology Korea University Seoul 02841 Republic of Korea; ^4^ Department of Mechanical Engineering Seoul National University Seoul 08826 Republic of Korea; ^5^ SK Hynix Co., Ltd. Incheon 17336 Republic of Korea; ^6^ Biomedical Engineering Research Center Asan Institute for Life Sciences Asan Medical Center College of Medicine University of Ulsan Seoul 05505 Republic of Korea; ^7^ Department of Integrative Energy Engineering Korea University Seoul 02841 Republic of Korea

**Keywords:** chronic biointerface, implantable electronics, laser processing, multiscale topography, topographical cue

## Abstract

Stable and reliable operation of implantable electronics must ensure both high‐quality electrical performance and chronic biocompatibility. Here, immune‐stealth implantable electronics fabricated by multiphoton ablation lithography are introduced. The cell‐repellent interface, consisting of micro‐grooves and nano‐islands, can be created by laser‐assisted topography patterning on a thin film substrate. This patterned surface demonstrates a 20‐fold increase in cell‐repellent effectiveness against immune cells such as macrophages and fibroblasts due to disturbance of focal adhesion. Furthermore, the cell‐repellent interface can also be patterned on the sub‐micron electrode layer without compromising its electrical and electrochemical performance. When the electrocardiogram (ECG) sensor applying the cell‐repellent interface is implanted into a rat subcutaneous tissue, inflammation and fibrotic reactions are effectively suppressed for 6 weeks. Consequently, stable ECG readings with clear PQRST waveforms are obtained in real‐time for 4 weeks, suggesting its potential to enhance chronic biocompatibility of implantable electronics.

## Graphical abstract

Immune‐stealth implantable electronics are developed using cell‐repellent interfaces created by multiphoton ablation lithography, which is the first research applying topographical cues on electronic devices. Laser‐patterned micro‐grooves and nano‐islands on ultrathin films can reduce immune cell adhesion to as low as 5%. The implanted sensors using topography are able to record stable electrocardiogram signals with reduced inflammation and fibrosis for 4 weeks, highlighting their promise for long‐term stable biomedical applications.

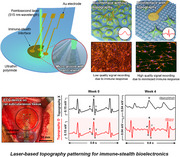

## 1 Introduction

Foreign body reaction (FBR) is a complex immune response triggered by the presence of exogenous materials in biological tissues.^[^ ^1^, ^2^, ^3^ ^]^ It is a fundamental mechanism of the human immune system, designed to recognize and respond to foreign substances through a cascade of inflammatory and fibrotic processes. While this response plays a crucial role in preserving tissue homeostasis and protecting against microbial invasion, it also poses significant challenges in the field of biomedical engineering. In particular, FBR is a major hurdle for the long‐term functionality of implantable devices, as the immune response often leads to fibrosis, encapsulation, and impaired device performance.^[^ ^4^, ^5^ ^]^ This issue is especially critical for implantable electronic devices designed for monitoring bioelectrical signals or performing therapeutic functions, as stable operation relies on maintaining effective electrical interfacing with surrounding tissues. Therefore, efforts to control and minimize FBR are highly required for enhancing the clinical applicability of implantable bioelectronics.^[^ ^6^, ^7^, ^8^, ^9^ ^]^

Various material modification strategies have been explored to mitigate the FBR associated with implantable electronic devices.^[^ ^10^ ^]^ One widely adopted approach involves reducing the mechanical mismatch between tissues and devices through the development of soft electronics, which are composed of ultrathin and deformable materials that closely mimic the mechanical properties of biological tissues.^[^ ^11^, ^12^, ^13^ ^]^ These soft electronic devices exhibit high flexibility, stretchability, and conformability, allowing them to integrate seamlessly with dynamic biological environments. By minimizing mechanical stress at the tissue‐device interface, soft electronics reduce tissue invasiveness and enable the acquisition of stable bioelectrical signals in vivo.^[^ ^14^, ^15^ ^]^ However, despite their improved mechanical compatibility, FBR remains a challenge, as immune cell adhesion and proliferation still occur at the implant site.

To further mitigate FBR and enhance the long‐term stability of implanted devices, surface modification strategies utilizing hydrophilic antifouling coatings have been introduced. The coatings form a dense hydration layer on the device surface, effectively preventing protein adsorption and subsequent immune activation. For instance, superhydrophilic zwitterionic hydrogel coatings applied to glucose sensors have been shown to inhibit fibrotic encapsulation for up to three months.^[^ ^16^ ^]^ Similarly, the controlled release of anti‐inflammatory drugs through crystallized drug formulations has demonstrated the ability to suppress fibrotic tissue formation around implanted glucose sensors for six months.^[^ ^17^ ^]^ However, these chemical strategies present challenges, including complex and time‐intensive synthesis processes and limited long‐term stability in mechanically dynamic tissue environments. Moreover, surface coatings may degrade the device's electrochemical performance by increasing interfacial impedance and interfering with charge transfer, ultimately hindering its ability to reliably record and transmit bioelectrical signals over time.

Considering the aforementioned strategies require strict material selection and time‐consuming processes for synthesis and fabrication, a more universal strategy that can be feasibly applied to different types of implantable electronics is necessary. From this perspective, topography modulation emerges as a promising candidate, as it allows for the regulation of the immune response simply by patterning the surface at the micro‐ and nanoscale, which has been actively studied at the in vitro level.^[^ ^18^, ^19^ ^]^ Numerous studies have demonstrated that engineered micro‐ and nanoscale topography can control cell behaviors such as adhesion, proliferation, differentiation, and migration.^[^ ^20^, ^21^, ^22^, ^23^, ^24^, ^25^ ^]^ However, designing topographical interfaces for flexible electronics has not yet been applied in vivo due to the limitations of the fabrication process. An appropriate surface engineering strategy that can be integrated with a well‐established fabrication system (e.g., photolithography) without compromising the electrical and mechanical properties of devices is urgently needed.

**Figure 1:**
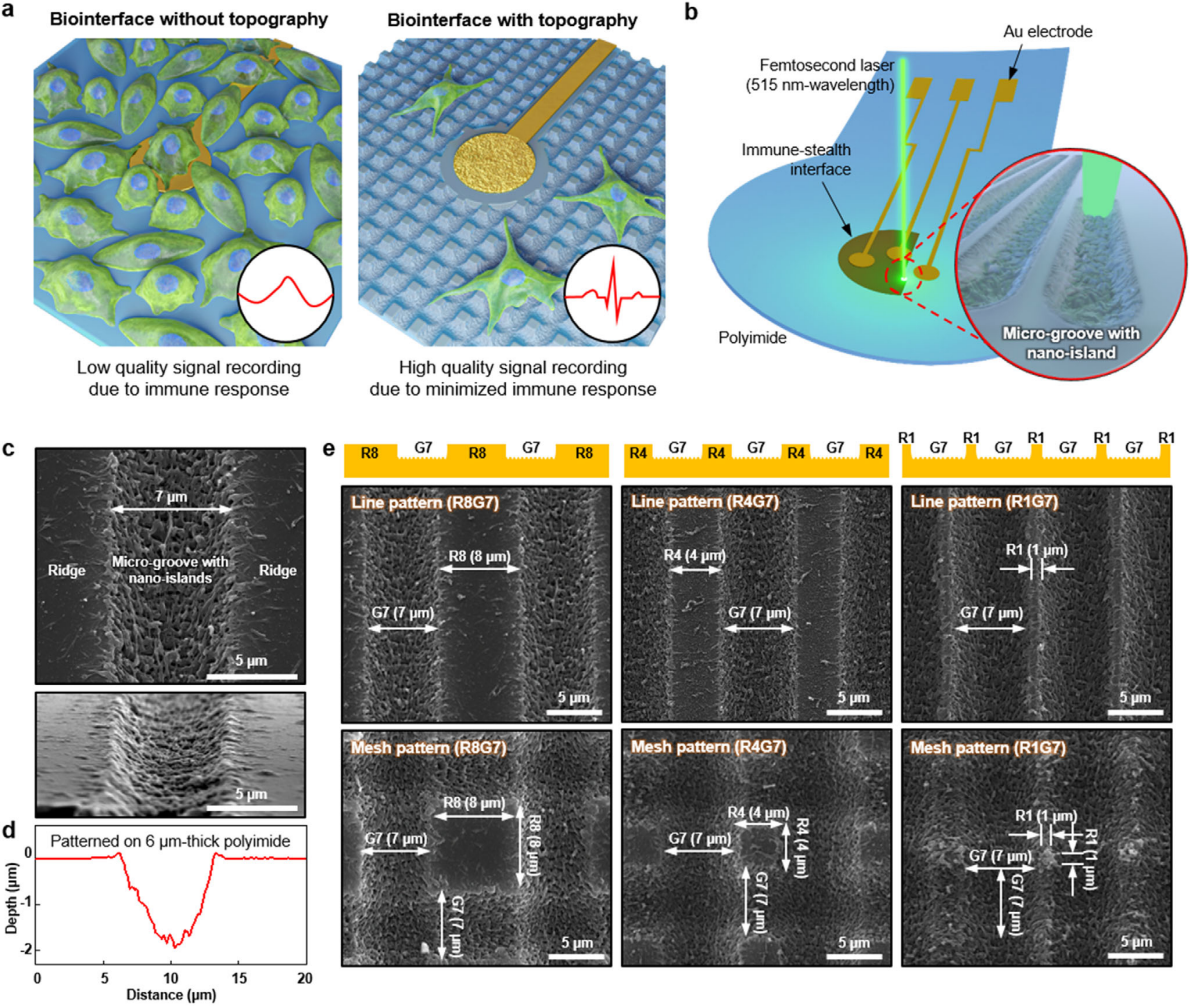
Topographical patterning of cell‐repellent interfaces for immune‐stealth electronics. a) Schematic illustration showing the immune responses at the biointerface without and with topographical patterning. b) Schematic illustration of multiphoton ablation lithography using femtosecond pulse laser. c) SEM images of polyimide thin film after multiphoton ablation lithography. The top image is the surface view and the bottom image is the tilted view. d) Depth profile of patterned polyimide, which is measured by using AFM. e) SEM images of patterned polyimide surfaces with various designs and spacings. R means ridge and G means groove.

Here, we present immune‐stealth implantable electronics enabled by the formation of a cell‐repellent interface on the surface of a thin film device. Using multiphoton ablation lithography, multiscale topography consisting of microscale grooves with nanoscale islands can be patterned directly on thin film electronics. This multiscale topography hinders immune cells from forming focal adhesions on the device surface, thereby reducing immune responses such as fibrosis. Thin film devices with the cell‐repellent multiscale topography can maintain signal fidelity due to the suppressed immune response, whereas devices without the cell‐repellent interface lose signal fidelity due to fibrosis (**Figure** **1**a). The cell‐repellent interface can be patterned directly on the device as a final step of the fabrication, which is achieved through a femtosecond laser‐induced ablation process (Figure 1b). There is no thermal stress and collateral material damage because heat exchange is minimized during laser pulse irradiation (≈400 fs). Under mechanically dynamic environments of tissues, the topographical interface applied to thin film electronics can sustain its immune‐stealth ability over a long time.

## 2 Results and Discussion

### 2.1 Multiscale Topography on Thin Film Substrate via Femtosecond Laser‐Based Multiphoton Ablation Lithography

Direct patterning of multiscale topography was performed on polyimide, one of the most widely used materials as a substrate and passivation layer for flexible electronics owing to its mechanical, thermal, and electrical stability. Scanning a femtosecond pulse laser on polyimide thin films induces the formation of microscale grooves with nanoscale islands (Figure 1c). Since the size of macrophages and fibroblasts before spreading is ≈10 µm,^[^ ^26^, ^27^ ^]^ the groove width is optimized as 7 µm. Because heat exchange is barely generated, laser‐assisted multiphoton ablation lithography can achieve resolutions at the scale of a few micrometers.^[^ ^28^ ^]^ While slower patterning speeds fully remove the whole polyimide film by ablation phenomenon, faster patterning speeds cannot render the line pattern but rather create successive circular structures with linear alignment (Figure S1a in the Supporting Information). Also, the width of the topography pattern can be modulated by controlling the laser irradiation energy (Figure S1b in the Supporting Information). Nanoscale islands are generated by directional resolidification of molten polyimide during the multiphoton ablation process, which results from the interaction between molten liquid polymer and gaseous products.^[^ ^29^ ^]^ Line profiles obtained by atomic force microscopy (AFM) show that the line pattern has a 7 µm‐width and 2 µm‐depth (Figure 1d; Figure S2 in the Supporting Information). Various patterns, including different ridge sizes and designs, can be created by adjusting the distance between two line patterns (Figure 1e). It is noted that R8G7 denotes repeated patterns of 8 µm wide ridges and 7 µm wide grooves. Furthermore, mesh shapes can also be patterned by scanning the laser both vertically and horizontally. Ridge sizes as small as 1 µm can be successfully patterned for both line and mesh designs. The laser patterning process causes a reduction in the thickness of the polyimide substrate, which may result in a decrease in mechanical strength.^[^ ^30^ ^]^ Although laser processing reduced the strain at the break of the polyimide substrate, the treated material still exhibited a higher strain capacity than native biological tissues,^[^ ^31^, ^32^ ^]^ including skin, subcutaneous tissue, and muscle, as shown in Figure S3 in the Supporting Information. This ensures that the device can endure mechanical deformations in vivo while simultaneously providing enhanced biocompatibility and reduced immune cell adhesion.

### 2.2 In Vitro Evaluation of the Cell‐Repellent Ability of Multiscale Topography

**Figure 2:**
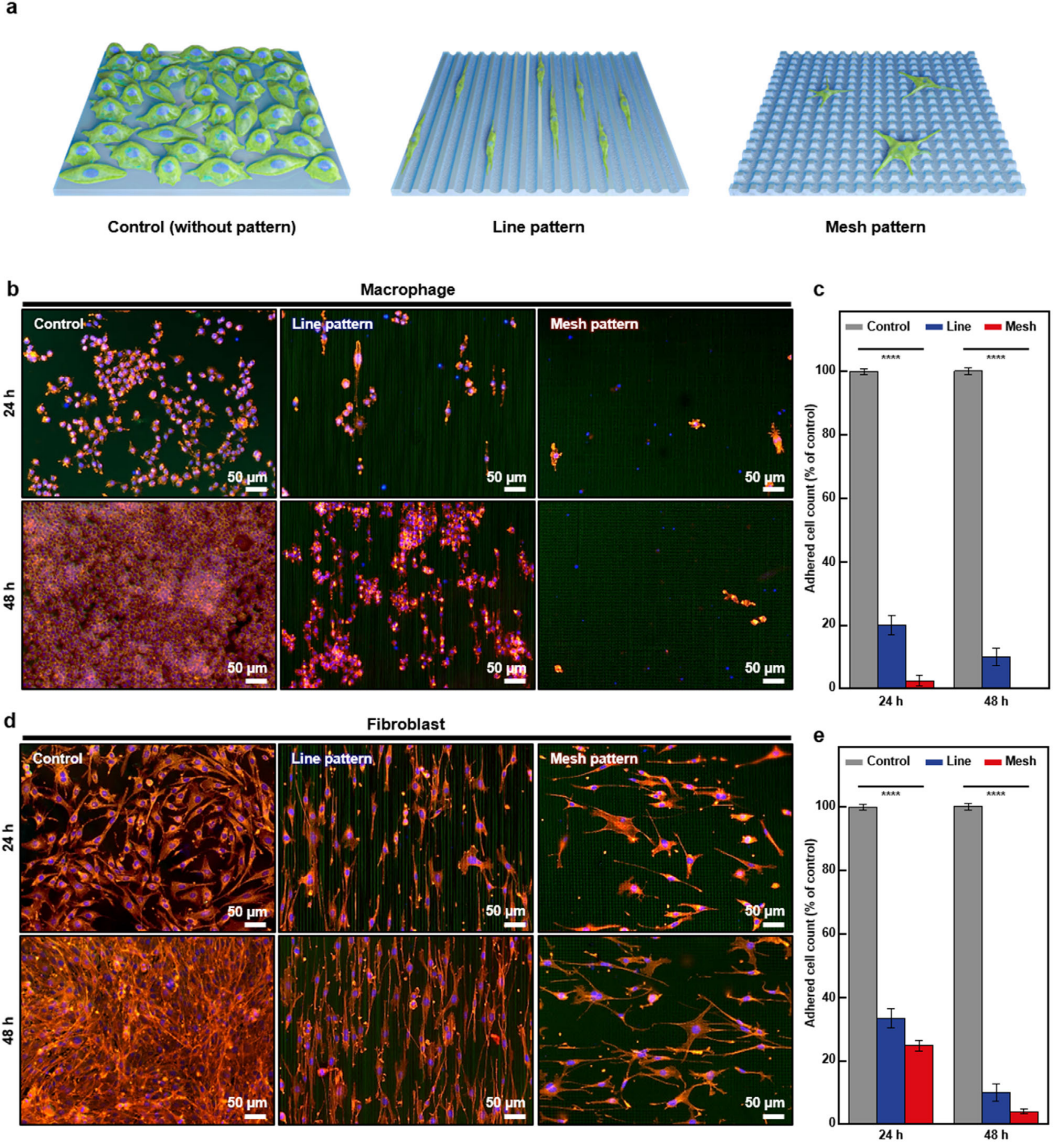
In vitro immune cell behaviors on polyimide thin film substrates without and with topographical patterning. a) Schematic illustration showing the difference in immune cell behaviors on various substrates including bare, line‐patterned (R1G7), and mesh‐patterned (R1G7) surfaces. b) Immunofluorescence image of cultured macrophages on various substrates at 24 and 48 h. c) Adhered macrophage cell count on various substrates at 24 and 48 h. Each value means the percentage of that of control. d) Immunofluorescence image of cultured fibroblasts on various substrates at 24 and 48 h. e) Adhered fibroblast cell count on various substrates at 24 and 48 h. Each value means the percentage of that of control.

To ensure the biocompatibility of the polyimide films before and after laser patterning, L929 fibroblasts were cultured in a medium containing both untreated (unpatterned) and laser‐patterned (mesh patterned, R1G7) polyimide films for 24 h. The results of the CCK‐8 cell viability assay showed that there was no significant cytotoxic effect for both types of polyimide films, confirming that the observed cell‐repellent ability was solely derived from the topographical modification rather than the cytotoxic effect of the materials (Figure S4 in the Supporting Information). Following this biocompatibility assessment, we investigated the immune cell behaviors associated with cell‐repellent multiscale topography in an in vitro environment. Immune cells were seeded on polyimide film substrates, including those not having patterns (control), those with R1G7 line patterns, and those with R1G7 mesh patterns (**Figure** **2**a). Since focal adhesions are typically formed within 12 h,^[^ ^33^, ^34^, ^35^ ^]^ the cells were observed for 48 h after seeding to assess adhesion and early proliferation. Macrophages, which play a major role in inducing immune responses at the acute stage of FBR,^[^ ^36^ ^]^ were seeded. When macrophages were placed on the bare polyimide substrate, they adhered stably because the flat surface of the polyimide thin film facilitates focal adhesion formation (the control group in Figure 2b,c). As a result, these adhered macrophages could proliferate without disruption. In contrast, macrophages seeded on the line‐patterned substrate struggled with adhesion due to multiscale topography created by multiphoton ablation lithography (line pattern group in Figure 2b,c). Repeated line patterns of microscale grooves with nanoscale islands hindered immune cells from forming focal adhesions. Although some macrophages hardly adhered to the patterned surface, they did not exhibit their typical round‐shaped morphology. Instead, they showed elongated or irregular star‐like morphologies as they attempted to form focal adhesions. Moreover, the immunofluorescence image of macrophages seeded on the line‐patterned film showed the meaningful expression of arginase 1, a marker of M2 macrophages that play an important role in anti‐inflammatory and immunosuppressive responses^[^ ^37^ ^]^ (Figure S5 in the Supporting Information). This result suggests that patterning the cell‐repellent interface can induce a phenotypic change in macrophages, potentially influencing regulatory effects such as signaling events.^[^ ^38^, ^39^ ^]^ Such changes can help resolve inflammation and promote the tissue healing process,^[^ ^40^, ^41^, ^42^ ^]^ which is necessary to be studied for more advanced immune‐stealth topographical strategies. Owing to the low number of adhered macrophages in the early stages, it is verified that cell density after 48 h of proliferation is considerably lower than that of the control group. Furthermore, the mesh‐patterned substrate exhibited superior cell‐repellent performance compared to the line‐patterned substrate (mesh pattern group in Figure 2b,c). This is because the mesh pattern provides insufficient surface area for the cell to form focal adhesions, whereas the line pattern leaves the non‐patterned area on ridge regions. Although 48 h is enough time to form focal adhesion,^[^ ^33^, ^34^, ^35^ ^]^ most macrophages were unable to adhere due to topographical interfaces.

To verify that the multiscale topography can repel immune cells associated with the chronic stage of FBR,^[^ ^43^ ^]^ the cell‐repellent effect against fibroblasts was also evaluated. Fibroblasts seeded on the bare polyimide substrate showed randomly oriented morphologies with stable adhesion (the control group in Figure 2d,e). Fluorescence image at 48 h showed that the bare substrate was covered by numerous fibroblasts. In contrast, the number of adhered fibroblasts on the line‐patterned substrate was 33.3% of that on the control group, meaning that multiscale topography also impedes focal adhesion formation of fibroblasts (line group in Figure 2d,e). Fibroblasts on the line‐patterned substrate exhibited elongated morphologies, corresponding to the direction of line patterns. In the case of the mesh‐patterned substrate, the number of adhered cells was much lower than that on the line‐patterned film as 24.7% of the control group, with fibroblasts exhibiting biaxially stretched morphologies. Although fibroblasts proliferated on the patterned substrates after 48 h, the density of adhered cells was much lower than in the case of the bare film, indicating that the cell‐repellent interface applied on polyimide substrate can effectively suppress the immune response in vitro. The cell‐repellent ability of different patterns, including R11G7 and R4G7, was also investigated to decide optimized patterning configuration (Figure S6 in the Supporting Information). Fewer macrophages and fibroblasts adhered to the line‐patterned substrates compared to the bare polyimide substrate. This is because the immune cells cannot easily form focal adhesions on surfaces with nanoscale roughness.^[^ ^44^, ^45^ ^]^ Interestingly, R4G7 shows more efficient repellency than R11G7. Considering the areal density of grooves, it is obvious that the patterning configuration with more grooves will have better repellency. Thus, we concluded that the optimal patterning configuration is R1G7. Numerous previous studies have shown that laser patterning on polyimide can synthesize graphene on its surface.^[^ ^46^, ^47^, ^48^ ^]^ To determine whether the cell‐repellent ability of topography pattern is a material effect or topographical effect, we analyzed the Raman spectroscopy of both bare and patterned polyimide thin film^[^ ^49^ ^]^ (Figure S7 in the Supporting Information). The Raman spectrum of patterned polyimide shows that laser‐induced graphene formation did not occur during multiphoton ablation lithography,^[^ ^50^ ^]^ as it generates minimized heat energy that is not enough to form laser‐induced graphene. Therefore, it can be concluded that the cell‐repellent ability is solely influenced by surface topography.

### 2.3 Multiphoton Ablation Lithography on Sub‐Micron Gold Electrode

**Figure 3:**
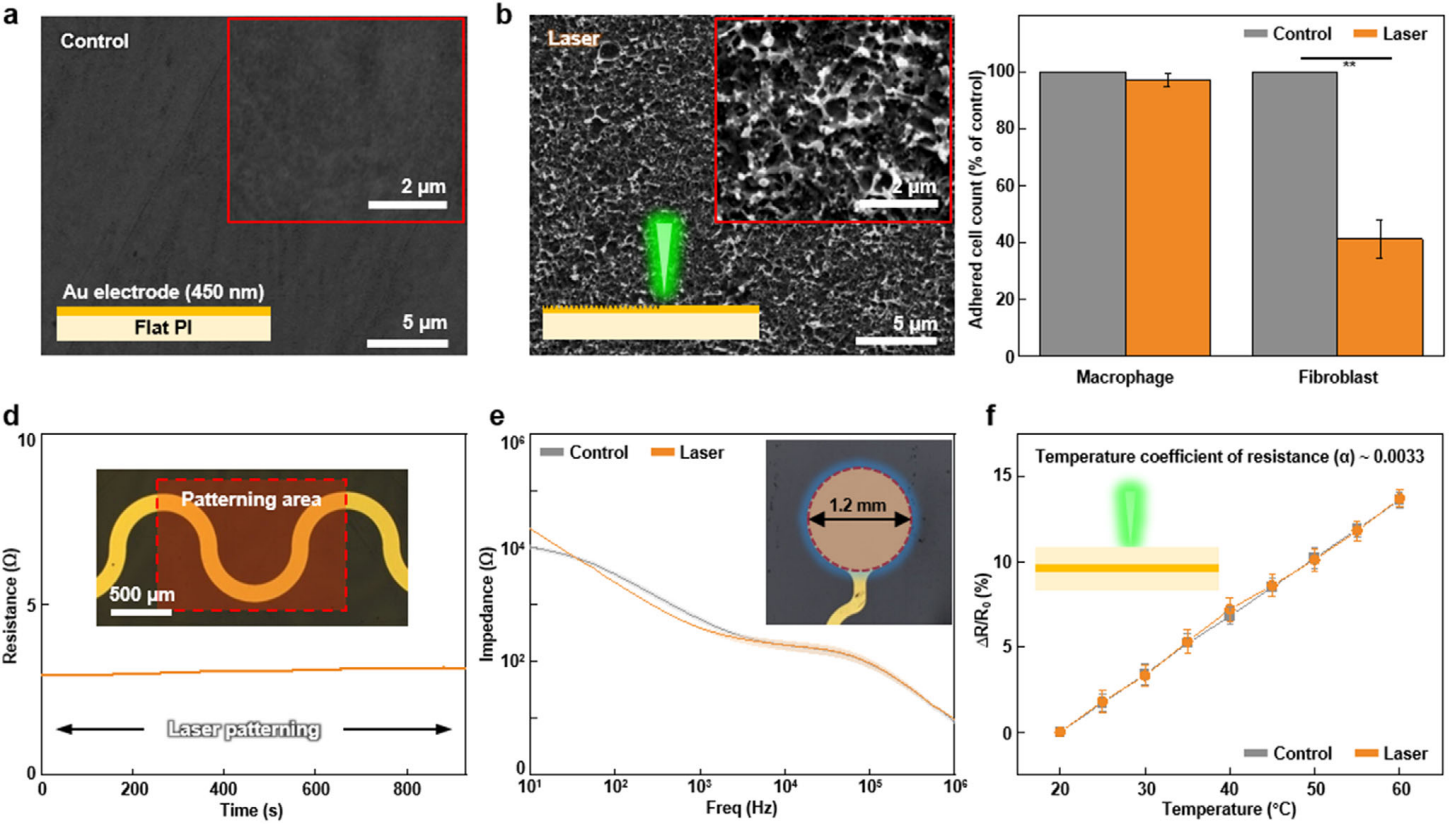
Multiphoton ablation lithography on Au thin film electrode. a) SEM image of Au electrode surface, which is deposited on polyimide thin film. The Inset image is a magnified view. b) SEM image of laser‐patterned Au electrode surface. The Inset image is a magnified view. c) Adhered immune cell count cultured on bare Au electrode (Control) and laser‐patterned Au electrode (Laser) at 24 h. Each value means the percentage of that of control. d) Real‐time resistance changes during multiphoton ablation lithography applied on the surface of Au electrode. The patterning process is conducted from 30 to 900 s. The Inset image shows the serpentine Au thin film electrode. e) Impedance of Au electrode without and with laser patterning in PBS solution at 37 °C (*n* = 3). f) Characteristics of Au resistor‐based temperature sensor without and with laser patterning (*n* = 3). The Inset scheme shows the cross‐section structure of the Au resistor‐based temperature sensor.

Since a conductive interface is a crucial component for implantable electronics to obtain clear electrophysiological signals, the surface of the conductive layer was also patterned to create an immune‐stealth interface using multiphoton ablation lithography. A 450 nm‐thick Au electrode was (1.2 mm in diameter) deposited on a 6 µm‐thick polyimide substrate using e‐beam lithography (**Figure** **3**a). When multiphoton ablation lithography was applied to the sub‐micron‐thick Au electrode layer, a rough surface with nanoscale islands was generated due to partial ablation of the Au surface (Figure 3b; Figure S8 in the Supporting Information). We verified that the patterned Au electrode can effectively repel the immune cells (Figure 3c). To distinguish the cell‐repellent effect from any material preference of cells, it is essential to verify that various immune cells do not inherently prefer either Au or polyimide. Therefore, macrophages and fibroblasts were seeded on an Au‐patterned polyimide substrate (Figure S9 in the Supporting Information). Seeded immune cells did not prefer the Au or polyimide surface and exhibited normal proliferation. For macrophages, there was no significant difference in the number of adhered cells between the bare Au electrode (Control) and the Au electrode with laser patterning (Laser). In contrast, the number of adhered fibroblasts on the Au electrode with the laser patterning is significantly lower than that on the bare Au surface. The immune cells are known to show different interactions depending on various substrates, which are influenced by the substrate's stiffness,^[^ ^51^, ^52^ ^]^ wettability,^[^ ^53^ ^]^ and surface charge.^[^ ^54^ ^]^ Therefore, the cell repellency of the patterned Au electrode may not be as remarkable as that of the patterned polyimide substrate. However, considering that the majority of tissue‐device interface consist of the substrate rather than the electrode, the degree of cell repellency on the patterned Au electrode may still be sufficient.

It is important to maintain original electrical performance even after the multiphoton ablation lithography process. Therefore, we measured the real‐time resistance change of the Au serpentine electrode during multiphoton ablation lithography using a digital multimeter. As a result, the resistance showed a negligible change during the laser patterning (Figure 3d). Additionally, it is important to ensure the electrochemical properties of the Au electrode even after multiphoton ablation lithography. Thus, we measured the impedance of two Au electrodes: the bare Au electrode and the Au electrode with laser patterning. There is no significant difference between the impedance curve of the bare and the patterned Au electrodes (Figure 3e; Figure S10 in the Supporting Information). The resulting impedance values of Au electrodes are sufficiently low for reliable electrophysiological signal acquisition.^[^ ^55^, ^56^, ^57^ ^]^ To examine the strong and weak points of overall surface processing technologies, we compared the electrochemical and biological properties of the laser‐treated electrodes with those of nanomaterial‐coated electrodes, as summarized in Table S1 in the Supporting Information. This comparison highlights the distinct advantages of individual strategies for surface modification. We also verified the applicability of multiphoton ablation lithography to a resistor‐based thin film temperature sensor composed of a polyimide substrate/encapsulation and an Au resistor (Figure S11 in the Supporting Information). When multiphoton ablation lithography was applied to the polyimide encapsulation, the patterned temperature sensor maintained its original temperature‐resistance characteristics (Figure 3f). This indicates that multiphoton ablation lithography can be applied to thin film electronics without compromising their original functions. As shown in Figure S12 in the Supporting Information, the charge storage capacity (CSC) of the Au electrodes remained stable after laser processing, exhibiting a slight increase from 8.67 × 10⁻⁷ to 9.04 × 10⁻⁷ C cm^−^^2^. This result confirms that laser patterning preserves the electrochemical performance of the treated electrodes while providing improved biocompatibility and long‐term signal stability.

### 2.4 In Vivo Evaluation of Implantable ECG Sensor with Immune‐Stealth Interface

**Figure 4:**
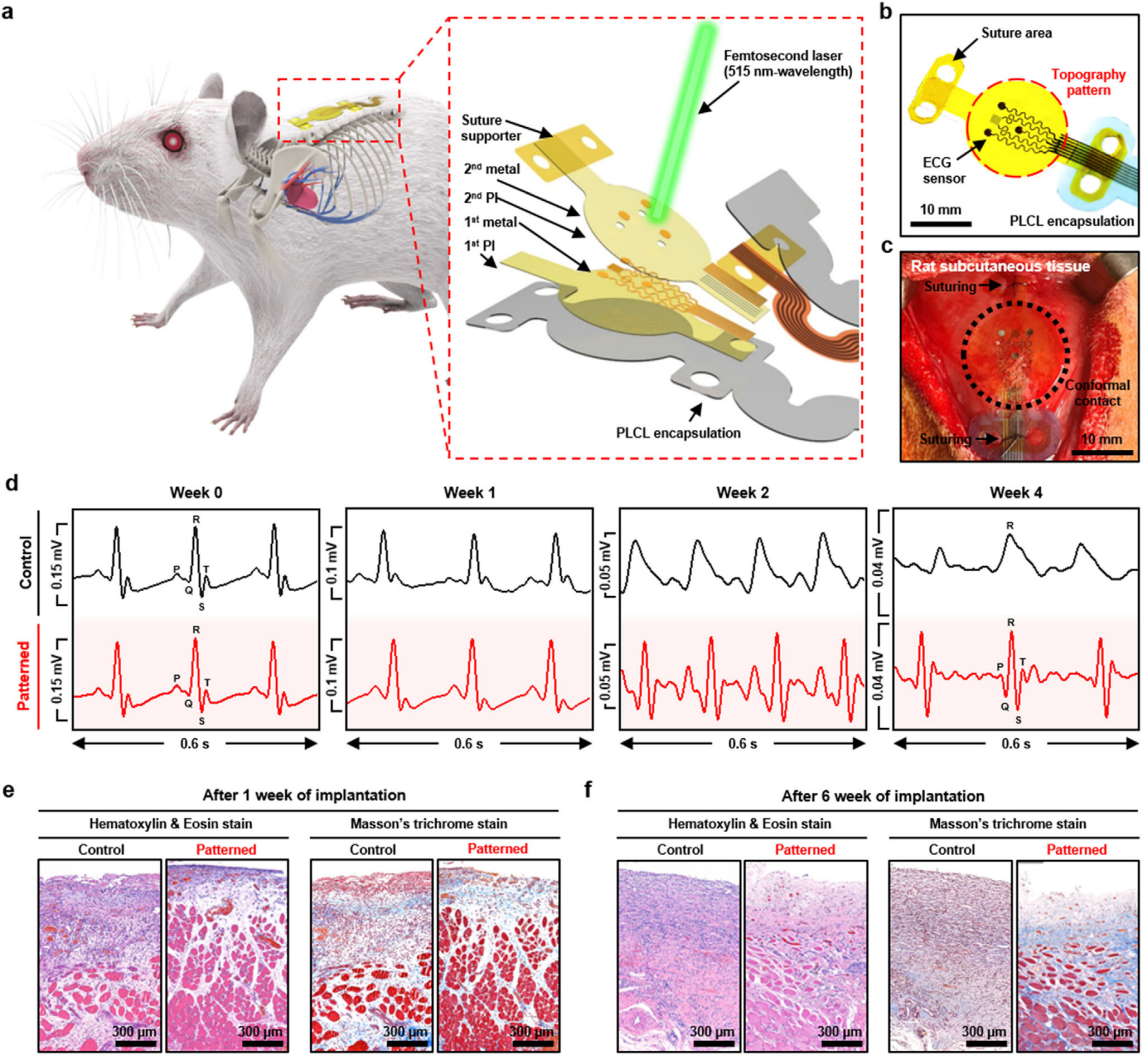
In vivo studies using immune‐stealth electronics achieved by cell‐repellent interfaces. a) Schematic illustration showing the immune‐stealth ECG sensor implanted on rat subcutaneous tissue. An expanded view of the immune‐stealth ECG sensor shows a multilayered thin film structure consisting of Au electrodes, polyimide substrate/encapsulation, and various supporting elements. b) Photograph of the immune‐stealth ECG sensor with topographical pattern. c) Photograph showing the implantation of an immune‐stealth ECG sensor on rat subcutaneous tissue. d) Recorded ECG signals from the implanted sensors without and with cell‐repellent topography pattern for 4 weeks. e, f) H&E staining and MT staining analyses of the rat tissue after the subcutaneous implantation of each ECG sensor for 1 week (e) and 6 weeks (f).

To investigate the effects of the cell‐repellent topography in vivo, it is essential to monitor a continuous and stable electrophysiological signal throughout the implantation period. Therefore, an immune‐stealth electronic device capable of ECG monitoring was implanted into the subcutaneous tissue of the rat's dorsal region, which is close to the heart (**Figure** **4**a). The device comprises a substrate and encapsulation layer both composed of a 7.5 µm‐thick polyimide film with the exposed Au electrodes. The cell‐repellent topography, R1G7 mesh pattern, was then created on the polyimide surface surrounding the Au electrodes, which formed an interface with the subcutaneous tissue (Figure 4b). Subsequently, the Au electrode was also patterned with its own cell‐repellent topography. To maintain a conformal tissue‐device interface even under dynamic mechanical strains induced by the rat's movements, additional structures were formed on both sides of the device, allowing for suturing (Figure 4c; Figure S13 in the Supporting Information). Devices with cell‐repellent interfaces (Patterned group) and without (Control group) were respectively implanted into the rats, and ECG signals were recorded for 4 weeks (Figure 4d). Specifically, the ECG signals obtained from the control group showed a crumbled PQRST waveform by week 2. After 4 weeks, only the R peak was distinguishable. This deterioration is attributed to the excessive localized deposition of ECM components, such as collagen, which electrically isolates cells and disrupt signal coherence, ultimately resulting in impaired resolution. In contrast, each P‐wave, QRS‐complex, and T‐wave could be clearly discriminated even after week 4, in the case of the pattered group. Considering that detailed analysis of the PQRST waveform should be feasible for the clinical utility of ECG signals,^[^ ^58^, ^59^, ^60^, ^61^, ^62^ ^]^ such results suggest that immune‐stealth electronics are applicable for chronic stable monitoring of bioelectrical signals. Furthermore, histological analyses using hematoxylin and eosin (H&E) staining and Masson's trichrome (MT) staining were conducted to observe the extent of immune responses at the tissue‐device interface (Figure 4d,e). One week after implantation, fibrotic reactions were significantly suppressed at the interface with cell‐repellent topography compared to that in the control group. In the control group, such inflammatory and fibrotic regions further expanded after 6 weeks of implantation compared to the first week. On the contrary, with the cell‐repellent interfaces, a mild level of immune response was maintained even after 6 weeks of implantation, which is similar to what was observed in the first week. To further deeply investigate the immune response process and develop ideal technology, it would be helpful to perform subsequent analyses of systemic immune responses such as the expression level of inflammatory cytokines.^[^ ^55^ ^]^ Additionally, Figure S14 in the Supporting Information demonstrates that temperature fluctuations at the cell‐repelling interface were relatively smaller than those in the control group, as observed through continuous measurements over a 4‐week period after implantation. These results obviously show that the immune response can be effectively suppressed at the cell‐repellent interface.

## 3 Conclusion

We present an immune‐stealth bio‐electronic interface enabled by the topographical engineering of a thin film electronic device. Leveraging the high‐resolution patterning ability of femtosecond pulse laser processing, the immune‐stealth interface consisting of repeated microscale grooves with nanoscale islands can be directly patterned on a polyimide thin film substrate. To evaluate the cell‐repellent ability of the laser‐assisted topography patterning in vitro, two immune cells, namely macrophages and fibroblasts, were seeded on the various patterned substrates. 48 h after seeding, the number of adhered cells on the immune‐stealth substrate was 20 times less than that of the flat substrate, owing to the disturbance of focal adhesion formation achieved by multiscale cell‐repellent topography. Furthermore, multiphoton ablation lithography was also applied to the sub‐micron‐thick Au electrode. Laser‐patterned Au electrode showed meaningful repellency against fibroblast, without compromising its electrical and electrochemical performance. Using this topographical engineering strategy, a polyimide thin film‐based ECG sensor with the immune‐stealth interface was implanted into the rat's subcutaneous tissue. After 4 weeks of implantation, the device with the immune‐stealth interface was enabled to obtain ECG signals that can clearly distinguish P‐wave, QRS complex, and T‐wave in real‐time. In contrast, the device without the immune‐stealth interface showed crumbled ECG signals that could not discriminate the PQRST wave. In addition, histological analyses were performed to investigate the extent of FBR at the implanted region. The results showed that the immune‐stealth interface can effectively suppress severe inflammation and fibrotic encapsulation, thus enabling stable and reliable recording of bioelectrical signals and chronically biocompatible existence in tissue. We expect that our technology will provide a useful strategy to achieve chronic implantable bioelectronics, which can potentially be expanded to clinical medical devices.

## 4 Experimental Section

### Preparation of Polyimide Thin Film

To make a polyimide thin film, poly(pyromellitic dianhydride‐co‐4 4′‐oxydianiline) amic acid solution (Sigma Aldrich) was deposited on a silicon wafer by spin coating. The condition of spin coating was 1000 rpm for 30 s. For polymerization, the silicon wafer was annealed on a hot plate with the following procedures: at 110 °C for 1 min, 150 °C for 15 min, and 250 °C for 2 h. The aforementioned process was repeated once more to ensure the polyimide film was thick enough not to be ablated by laser.

### Patterning of Cell‐Repellent Interfaces Using Multiphoton Ablation Lithography

To enable multiphoton ablation lithography, a 1030 nm wavelength Yb:KYW femtosecond laser (s‐pulse HP, Amplitude, Pessac, France) was used, manually adjusted to ensure a 515 nm wavelength. The laser provided a pulse duration of 400 fs, repetition rates of 1 kHz, and peak energy of 7 µJ. A 10× lens and neutral density filter (Thorlabs, NE10A) were used to reduce laser beam diameter and power. For pulse power measurement, a low‐power thermal sensor connected was used to a power meter (Nova II, Ophir). Multiscale topography on polyimide substrate was patterned using 300 nJ laser energy and 0.1–10 mm s^−1^ laser scan speed. The optimum laser scan speed for cell‐repellent multiscale topography on polyimide was 1 mm s^−1^. The actual interval distance of R11G7, R8G7, R4G7, and R1G7 between two grooves was 18, 15, 11, and 8 µm. The laser patterning conditions for both line pattern and mesh pattern were identical. Multiscale topography on Au thin film was patterned using 100 nJ. The optimum laser scan speed for cell‐repellent multiscale topography on Au electrode was 1 mm s^−1^. The actual interval distance of the laser‐patterned Au electrode was 1 µm. After patterning, mild sonication treatment was conducted to remove adhered debris from the film.

### Surface Analysis Using SEM and AFM

Field‐emission scanning electron microscopy (FE‐SEM; Inspect F50, FEI) was used to analyze the surface microstructure of the polyimide films under various patterning conditions. To analyze the surface profile of the polyimide films, atomic force microscopy (AFM; XE‐100, Park Systems) was used with 20 × 20 µm scan size, noncontact mode, and an NCHR cantilever.

### Cell Culturing and Immunofluorescence Imaging of Adhered Cells on Various Substrates

Macrophages and fibroblasts were used to evaluate the effect of focal adhesion disturbance on the surface of the thin film device in vitro. The mouse macrophages (RAW264.7, ATCC) and mouse fibroblasts (L929, ATCC) were seeded on each specimen surface and incubated for 24 and 48 h in Dulbecco's modified Eagle's medium (DMEM, Welgene) solution supplemented with 10% fetal bovine serum (FBS, Gibco) and 1% penicillin (Gibco) at 37 °C, 5% CO₂ and 95% humidity. Cells that did not attach to the surface were removed by washing three times with PBS. Cells attached to the specimens were fixed with 4% formaldehyde (4% paraformaldehyde in PBS) for 5 min and washed three times with PBS. Subsequently, F‐actin was stained with rhodamine‐phalloidin (R415, Invitrogen), and the nucleus was stained with DAPI (LS‐J1033, Vectashield) and observed using a fluorescence microscope (Axioscope imager A2M, ZEISS). The images taken at different locations of each specimen were analyzed using the ImageJ program (NIH) to determine the number of cells per unit area.

### Cell Immunofluorescence Analysis

The expression of Arginase‐1 was analyzed by immunohistochemistry. After culturing the macrophages on the surface of the thin film device, the culture medium was removed and rinsed three times with PBS to remove the excess, and 4% paraformaldehyde solution was added and fixed for 20 min. Subsequently, the cell culture plate was rinsed three times with PBS, and permeabilized in 0.5% Triton X‐100 PBS solution for 5 min. After rinsing three times with PBS solution, 1% FBS solution was added and blocking was performed for 30 min. The cytoplasmic staining was performed with rhodamine phalloidin (R415, Invitrogen) and the primary antibody was Arginase‐1 antibody (9819, Cell Signaling Technology). The secondary antibody was Alexa fluor 488 phalloidin (A12379, Invitrogen). All cells were stained with DAPI solution in the mounting medium for fluorescence (H‐1200, Vector) prior to imaging, and images were obtained by fluorescence microscopy (Axioscope imager A2M, ZEISS).

### Cytotoxicity Test

The cytotoxicity test was conducted in accordance with the ISO 10993‐12 protocol. L929 fibroblasts were seeded in a 96‐well plate at a density of 1 × 10^5^ cells per well and incubated at 37 °C under physiological conditions. After 24 h, the culture medium was replaced with fresh medium containing a polyimide film (6 mm^2^ in size), and the cells were further incubated for an additional 24 h. Subsequently, 10 µL of CCK‐8 solution was added to each well containing 100 µL of phenol red‐free medium, followed by incubation at 37 °C for 2 h. Absorbance was measured at 450 nm using a microplate spectrophotometer (Biomax Discover System, Promega) to determine cell viability. The percentage of cell viability was calculated using the following formula:

(1) $$\mathrm{Cell}\;\mathrm{viability}\;\left(\%\right)=\frac{\left(OD_{Sample}-OD_{Blank}\right)}{\left(OD_{Control}-OD_{Blank}\right)}\times 100$$

### Preparation of EP Sensor and Temperature Sensor on Polyimide Thin Film

A substrate was prepared by spin coating a bilayer of polyimide ≈6 µm thick) on a Si wafer (*p*‐type, Silicon Technology Co., Japan). The Au electrodes (Cr 5 nm/Au 450 nm) were deposited and patterned on the substrate by e‐beam evaporation and photolithography. After spin‐coating a layer of PI 6 µm thick) as a flexible insulator, it was formed. Dry reactive ion etching (RIE, JVAC, South Korea) was used to define the opening for electrical contact pads of the Au electrodes and external interfaces.

### Measurement of Impedance

The electrical impedance of various nanocomposite conductors was evaluated using electrochemical impedance spectroscopy (CH Instruments, CHI6602). The Au electrode (working electrode) was connected using a copper wire with silver paste. The Ag/AgCl (reference electrode) and counter electrode (Pt electrode) were immersed in PBS solution contained within a manually fabricated PDMS wall at the exposed Au electrode area. The measurements were performed in pH 7.4 PBS solution (Welgene). The exposed area of the Au electrode was designed to be Ø 1.2 mm.

### Measurement of Resistance

The resistance of various Au electrodes was measured using a digital multimeter (Keithley, DMM6500). First, each contact pad is connected to an external copper wire using commercial silver paste in order to measure the real‐time resistance change during multiphoton ablation lithography.

### Fabrication of Thin Film‐Based Implantable Electronic Device

A temporary substrate was prepared by spin coating a bilayer of poly(methylmethacrylate) (PMMA, MicroChem, USA; ≈100 nm thick) and polyimide (≈6 µm thick) on a Si wafer (p‐type, Silicon Technology Co., Japan). The bottom electrodes (Cr 5 nm/Au 450 nm) were deposited and patterned on the substrate by e‐beam evaporation and photolithography. After spin‐coating a layer of diluted PI (≈6 µm thick) as a flexible insulator, dry reactive ion etching (RIE, JVAC, South Korea) was used to create openings for the electrical contact pad. Another layer of Au was deposited to form the contact pads of the top electrodes, and RIE was used to define the circular device shape. Removal of the PMMA layer by immersing the substrate in acetone enabled the release of the device, and a suture supporter made of PI tape was attached to the top and bottom parts of the device. After bonding an ACF cable to the exposed electrodes to interface with an Arduino microcontroller, a prefabricated film of PLCL (140k, ≈50 µm thick) was placed on the bottom of the device for encapsulation.

### Implantation of Thin Film‐Based Implantable Electronic Device

All animal experiments were approved by the Institutional Animal Care and Use Committee (approval number: IACUC‐2019‐14‐036), Asan Medical Center, and followed the ethical principles for animal experimentation established by the institute. A total 20 Sprague‐Dawley rats (male, 8 weeks old, 250 g) were used that were purchased from Orientbio (South Korea). For implantation of the thin film‐based implantable electronic device, animals were anesthetized with Zoletil (50 mg kg^−1^) and xylazine (10 mg kg^−1^) intramuscularly. The animals were placed in the prone position, and their dorsal hair was removed and disinfected with povidone‐iodine. The subcutaneous tissue was exposed through a skin incision. The device was sutured using 4‐0 silk sutures (AILEE, South Korea). The skin was aseptically closed using 2‐0 silk sutures (AILEE, South Korea) and scrubbed with povidone‐iodine solution. To alleviate pain and prevent infection, antibiotics (50 mg kg^−1^, ampicillin, Samyang Anipharm, South Korea) and analgesics (5 mg kg^−1^, ketorolac tromethamine, DongKwang pharm, South Korea) were administered intramuscularly once daily for 3 days after the surgery.

### Electrocardiogram Measurement

The ECG monitoring device was connected to an external manipulation system. Using a customized software program (LabVIEW 2015, National Instruments, USA), ECG signals were measured in real‐time. Collected signals were recorded by source meter (Keithley 2636B, USA) and were amplified by differential AC amplifier (Model 1700, A‐M Systems, USA). Then, individual datasets were collected by a DAQ system (USB‐ 6255, National Instruments, USA) with a sampling frequency of 1 kHz.

### Histological Analysis

ECG monitoring devices with and without cell‐repellent interfaces were implanted into the subcutaneous tissue of the rat's dorsal region. The rats were sacrificed at 1 week and 6 weeks after implantation. Tissues were harvested from the region in contact with the device, and the obtained tissue samples were fixed with 4% paraformaldehyde in PBS for 2 days and embedded in paraffin. The paraffin sections were stained with H&E and MT by following the standard protocol.

### Statistical Significance Analysis

All data were analyzed using a one‐way analysis of variance (ANOVA) with Tukey's post hoc test and are expressed as the mean ± standard deviation. The significance levels were set as follows: NS (not significant) *p* > 0.05, ^*^ *p* < 0.05, ^**^ *p* < 0.01, ^***^ *p* < 0.001, and ^****^ *p* < 0.0001.

## Conflict of Interest

The authors declare no conflict of interest.

## Author Contributions

H.S., G.‐J.K., S.S., and J.H.L. contributed equally to this work. Y.S., S.H., C.‐H.E., S.K., and K.L. conducted in‐vitro experiments. H. Kim and K.‐S.L. supported in‐vivo experiments. S.H., C.‐H.E., Y.‐C.K., H.K., S.K., and S.‐E.M. supported data analysis. H.S., G.‐J.K., S.S., S.H.K., S.‐W.H., and H.J. wrote the manuscript. S.H.K., S.‐W.H., and H.J. supervised this work. All authors read and approved the final manuscript. The authors declare no competing interests.

## Supporting information



Supporting Information

## Data Availability

The data that support the findings of this study are available from the corresponding author upon reasonable request.
