# Supplementary material for: Topographical Patterning of Cell‐Repellent Interfaces for Immune‐Stealth Implantable Electronics via Multiphoton Ablation Lithography
Source: Adv Sci (Weinh). 2025 Jun 19;12(34):e06482. doi: 10.1002/advs.202506482 (PMC12442681; doi:10.1002/advs.202506482)
Supplement: Supplementary file 1 — Supporting Information [file ADVS-12-e06482-s001.docx]

Supporting Information

Topographical patterning of cell-repellent interfaces for immune-stealth implantable electronics via multiphoton ablation lithography

Hyunseon Seo, Gwan-Jin Ko, Sangmin Song, Joong Hoon Lee, Youngmin Seo, Sungkeun Han, Chan-Hwi Eom, Hyewon Kim, Seongsoo Kim, Kang-Sik Lee, Yu-Chan Kim, Hojun Kim, Si-Eun Moon, Kyungwoo Lee, Seung Hwan Ko*, Suk-Won Hwang*, Hojeong Jeon*


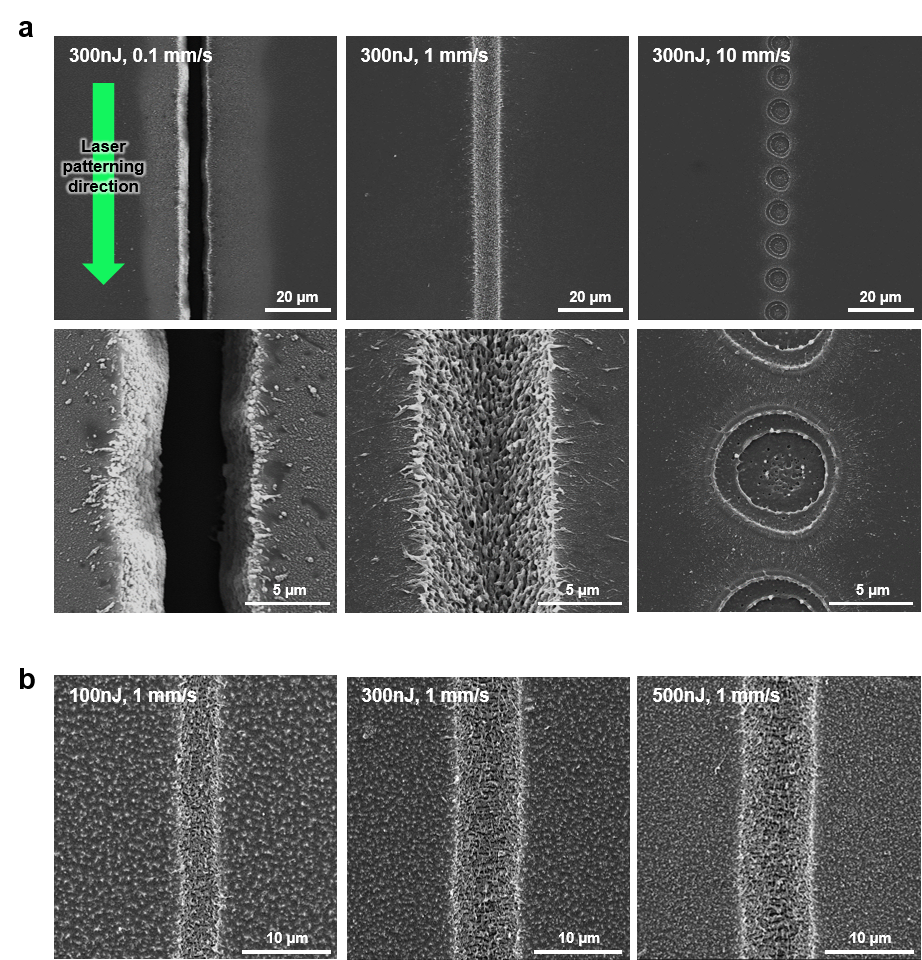


**Figure S1.** SEM image of polyimide surface after laser patterning with various a) scanning speeds and b) pulse energies.


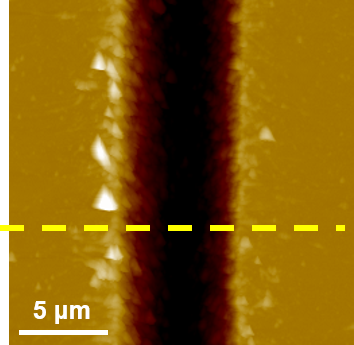


**Figure S2.** AFM image of polyimide surface after laser patterning. Dotted yellow line is line profile path of Fig. 1e.


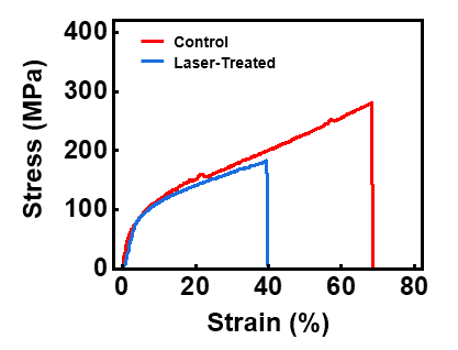


**Figure S3.** Comparison of tensile test (mechanical strength of implantable devices) results before and after laser treatment.


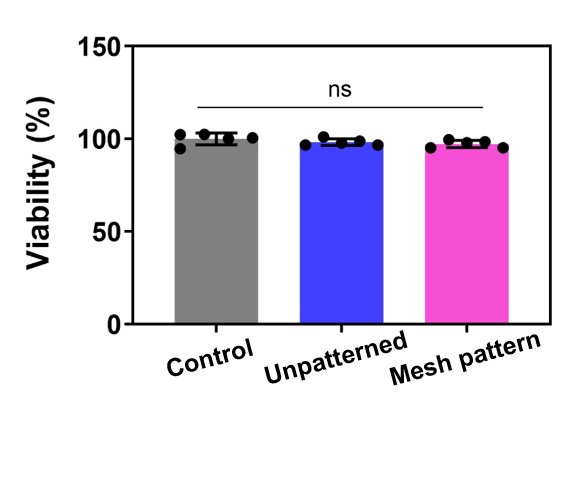


**Figure S4.** Cytotoxicity of polyimide thin films without pattern and with mesh pattern (R1G7).


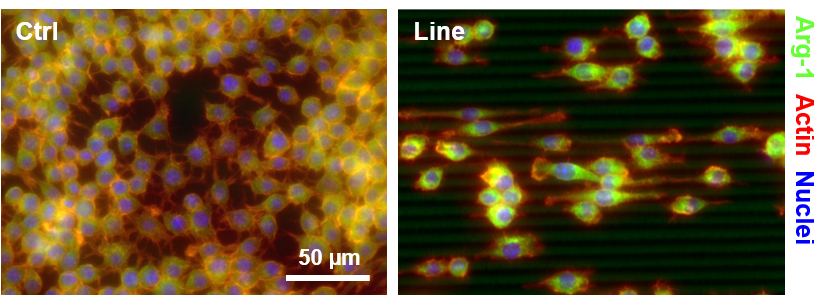


**Figure S5.** Immunofluorescence image of macrophages seeded on the bare polyimide film and line-patterned polyimide film. Macrophages on the line-patterned film shows expression of M2 state (Arg-1).


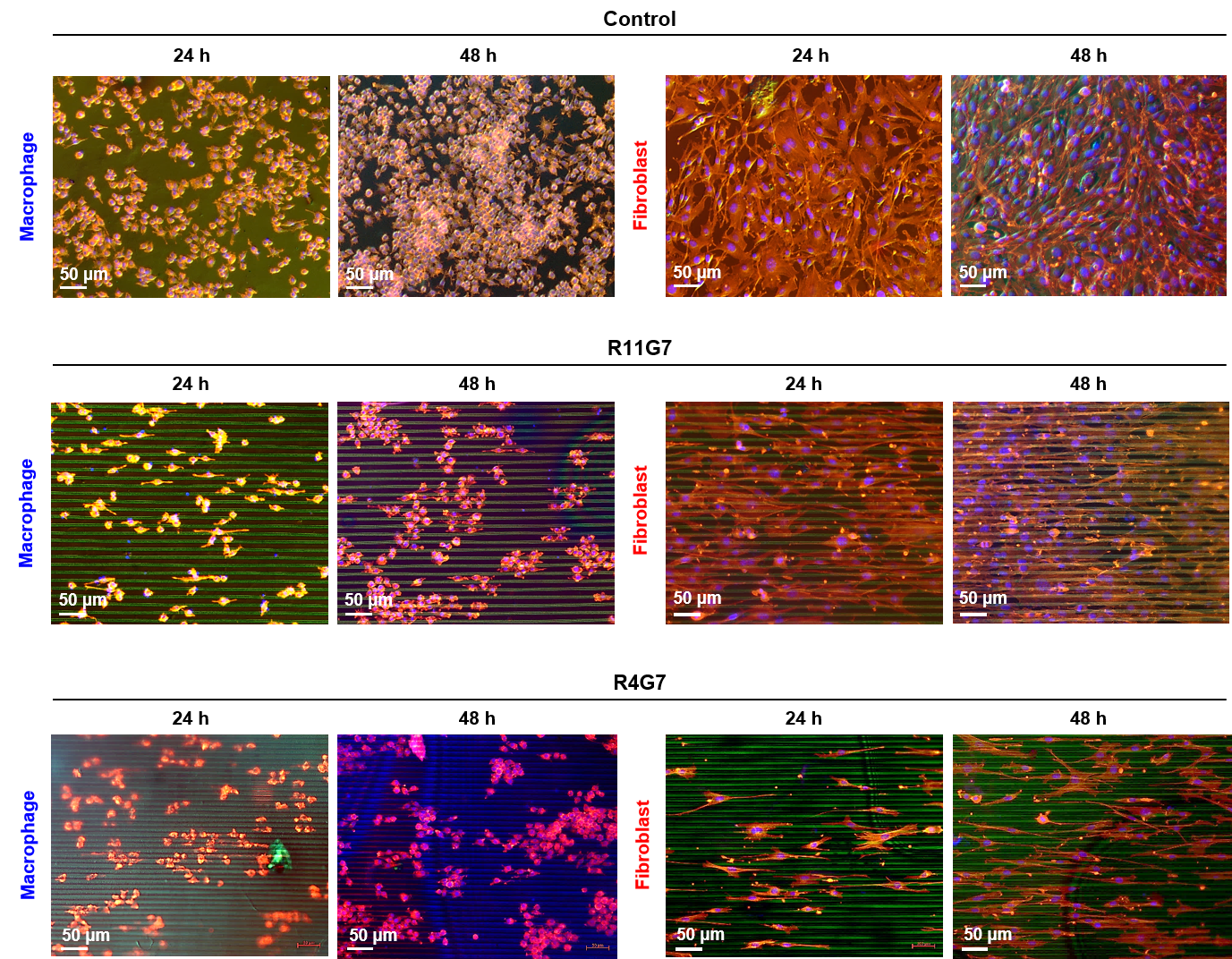


**Figure S6.** Immunofluorescence image of immune cells seeded on bare polyimide film, line patterned polyimide film with R11G7 (Ridge 11 μm and groove 7 μm), and R4G7 (Ridge 4 μm and groove 7 μm)


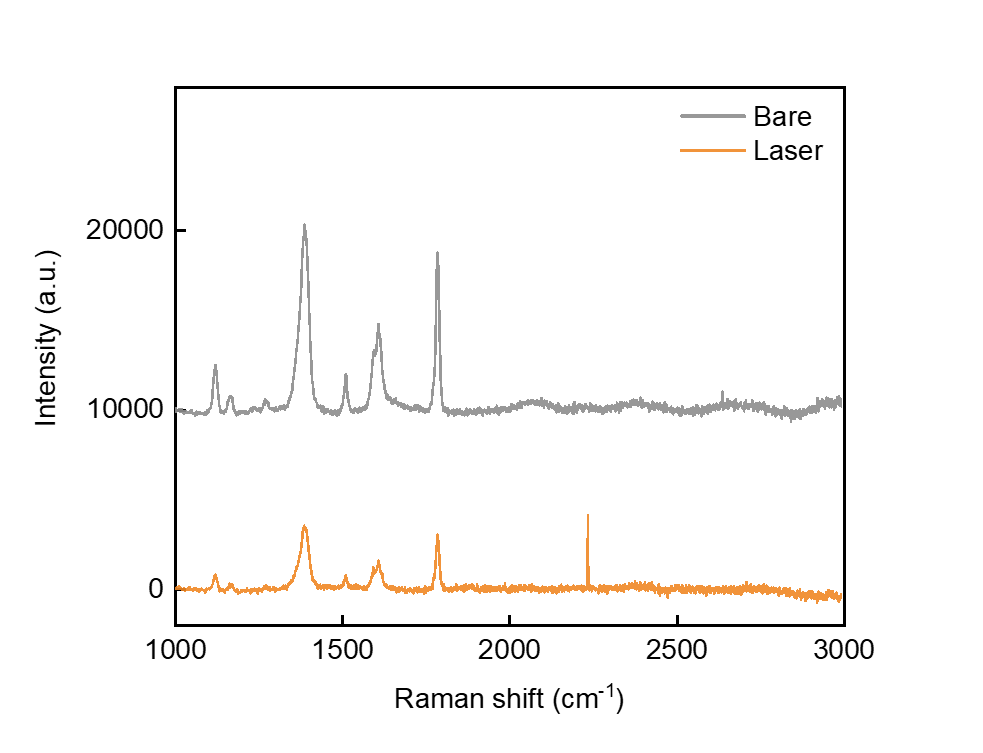


**Figure S7.** Raman spectroscopy of polyimide surface without and with laser patterning. Considering that two curves are similar and graphene peaks are not observed, multiphoton ablation lithography does not synthesize graphene on polyimide surface.


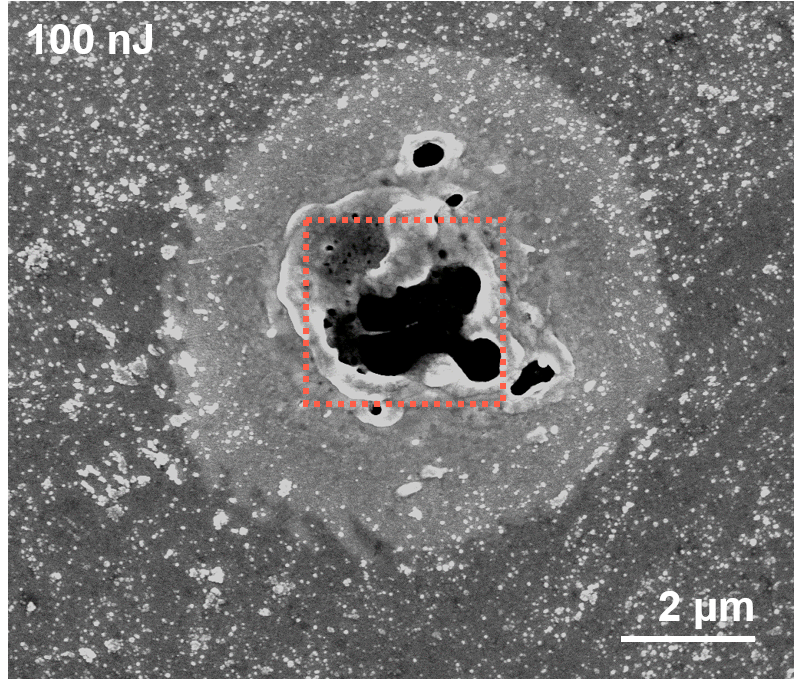


**Figure S8.** Surface image of 100 nm-thick gold film after single pulse laser ablation. Since stable laser irradiation is challenging to achieve with laser energy lower than 100 nJ, we set 100 nJ as the minimal energy threshold to ensure reliable reproducibility. At this energy level, we observed that a 100 nm-thick gold film undergoes damage due to laser-induced ablation. Therefore, to enable topographical patterning without surface ablation, it was necessary to fabricate a thicker gold film (450 nm-thick).


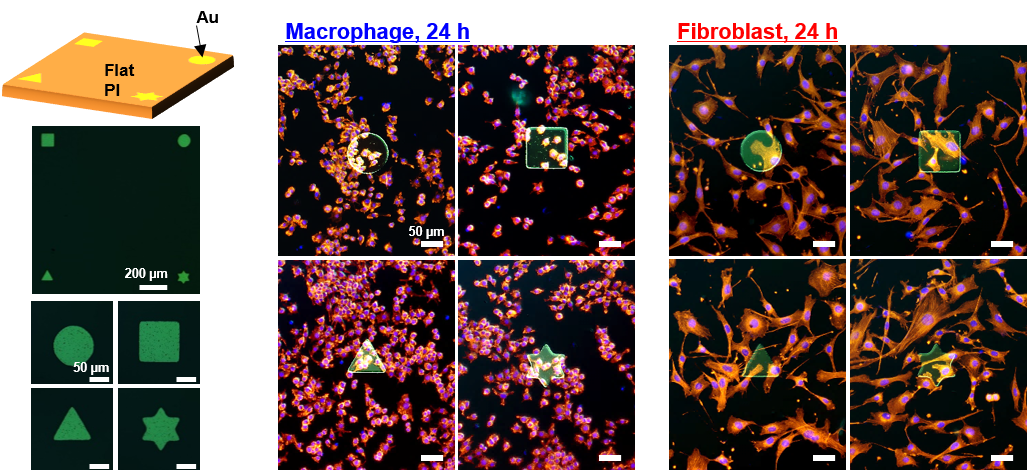


**Figure S9.** Verifying experiment of the material preference of macrophage and fibroblast. Immune cells were seeded on polyimide film with Au pattern. Immunofluorescence image shows that there is no preference to either polyimide or Au.


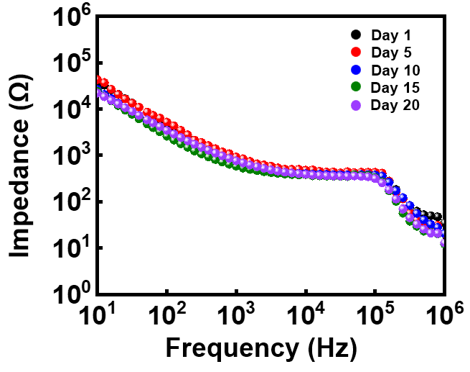


**Figure S10.** Changes in impedance of the later-treated Au electrode at room temperature as a function frequency (1 to 10^6^ Hz) over a period of up to 20 days.


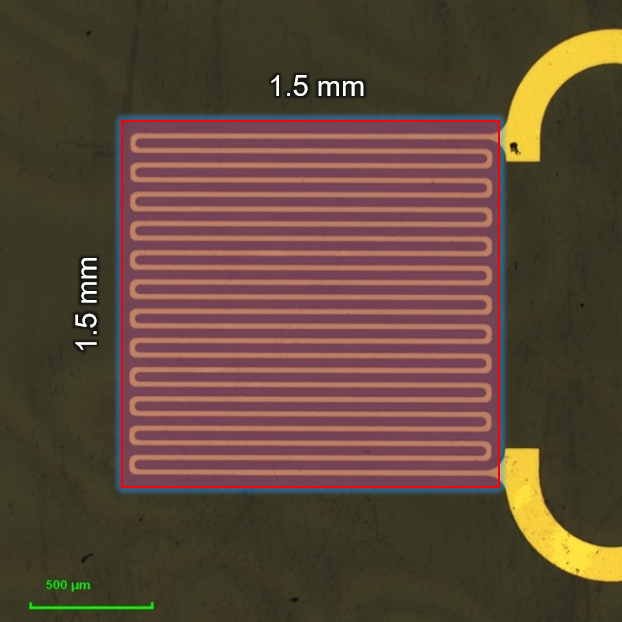


**Figure S11.** Design and structure of Au resistor-based temperature sensor. Red colored area is subjected to laser patterning.


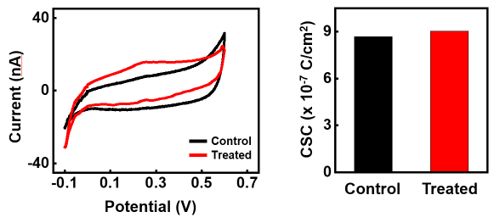


**Figure S12.** Cyclic voltammetry (CV) curves and charge storage capacitance (CSC) comparison of the Au electrodes with and without laser treatment.


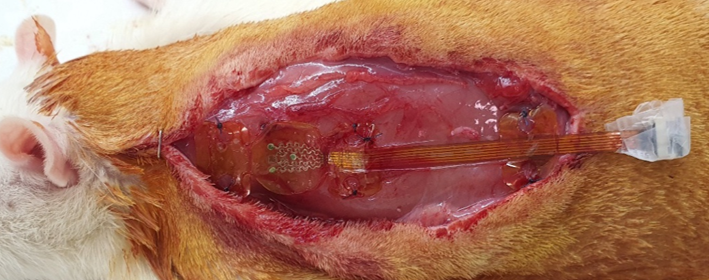


**Figure S13.** Photograph of ACF cable connected to in-vivo device. To ensure stable long-term implantation, the terminal part of the ACF cable was wrapped with sterile tape and implanted subcutaneously in the rat. To minimize excessive movement of the ACF cable under the subcutaneous tissue, we incorporated an additional suture area at the terminal region for secure fixation. For each measurement, a small incision was made in the subcutaneous tissue to expose the terminal part, allowing it to be connected to the external DAQ system before conducting the measurement. This experimental design enabled multiple weeks of stable ECG recording while maintaining the implant’s integrity. Importantly, this design ensured that the ECG sensor part, which interfaces directly with the tissue, remained undisturbed, allowing for stable and reliable long-term signal recording without affecting the tissue-device interface.


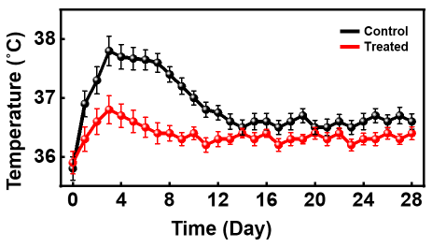


**Figure S14.** Continuous measurement of temperature changes over a 4-week period following subcutaneous implantation of immune-stealth temperature sensor.


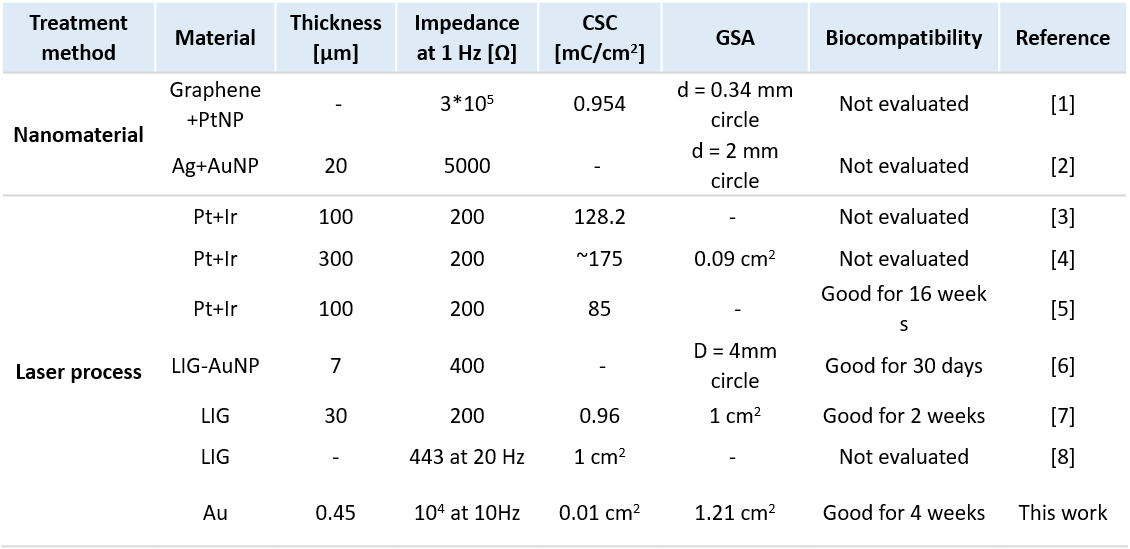


**Table S1.** Comparison of surface modification method between nanomaterial coating and laser processing.

**References in Supporting Information**

[1] Babaroud, Nasim Bakhshaee, et al. "Surface modification of multilayer graphene electrodes by local printing of platinum nanoparticles using spark ablation for neural interfacing." *Nanoscale* 16.7 (**2024**): 3549-3559.

[2] Matvieiev, Oleksandr, et al. "Effect of different modification by gold nanoparticles on the electrochemical performance of screen-printed sensors with boron-doped diamond electrode." *Scientific Reports* 13.1 (**2023**): 21525.

[3] Li, Linze, Changqing Jiang, and Luming Li. "Hierarchical platinum–iridium neural electrodes structured by femtosecond laser for superwicking interface and superior charge storage capacity." *Bio-Design and Manufacturing* 5.1 (**2022**): 163-173.

[4] Amini, Shahram, et al. "Femtosecond laser hierarchical surface restructuring for next generation neural interfacing electrodes and microelectrode arrays." *Scientific Reports* 12.1 (**2022**): 13966.

[5] Li, Linze, et al. "Electrochemical and biological performance of hierarchical platinum-iridium electrodes structured by a femtosecond laser." *Microsystems & nanoengineering* 8.1 (**2022**): 96.

[6] Huang, Xingcan, et al. "Transient, implantable, ultrathin biofuel cells enabled by laser-induced graphene and gold nanoparticles composite." Nano Letters 22.8 (**2022**): 3447-3456.

[7] Zhao, Lei, et al. "Robust, stretchable bioelectronic interfaces for cardiac pacing enabled by interfacial transfer of laser-induced graphene via water-response, nonswellable PVA gels." *Biosensors and Bioelectronics* 261 (**2024**): 116453.

[8] Yang, Jie, et al. "Facile fabrication of robust and reusable PDMS supported graphene dry electrodes for wearable electrocardiogram monitoring." *Advanced Materials Technologies* 6.9 (**2021**): 2100262.
